# Analyzing a national health surveillance strategy to reduce mother-to-child transmission of syphilis: The case of Brazilian investigation committees

**DOI:** 10.1016/j.ijregi.2023.07.008

**Published:** 2023-08-10

**Authors:** Thereza Cristina de Souza Mareco, Thaísa Gois Faria de Moura Santos Lima, Maria Natália Pereira Ramos, Marquiony Marques dos Santos, José Adailton da Silva, Vania Priamo, Cintia Michele Gondim de Brito, Esdras Daniel dos Santos Pereira, Carlos Alberto Pereira de Oliveira, Lyane Ramalho Cortez, Ion Garcia Mascarenhas de Andrade, Milena Cristina Duarte de Almeida, Ricardo Alexsandro de Medeiros Valentim

**Affiliations:** a Laboratory for Technological Innovation in Health, Federal University of Rio Grande do Norte, Natal, Brazil; b University of Brasília, Brasília, Federal District, Brazil; c Open University of Portugal, Lisbon, Portugal; d Graduate Program in Health Management and Innovation, Federal University of Rio Grande do Norte, Natal, Brazil; e University of Pernambuco, Recife, Brazil; f Oswaldo Cruz Foundation, Rio de Janeiro, Brazil; g State University of Rio de Janeiro, Rio de Janeiro, Brazil

**Keywords:** Mother-to-child transmission of infectious diseases, Congenital syphilis, Public health policy

## Abstract

**Objectives:**

This study aimed to analyze the relevance of investigation committees in eliminating mother-to-child transmission of syphilis in Brazil.

**Methods:**

Questionnaires and interviews were conducted with health managers of 25 Brazilian Federative Units and Brazil's Federal District. Data were analyzed using Bardin's content analysis technique and subsequently compared with the global prescriptions for syphilis response of the Pan American Health Organization, World Health Organization, and recent research publications examining the course of syphilis in Brazil, in Brazilian regions, and globally.

**Results:**

While the investigation committees drew on the successful experience of those in reducing maternal mortality, which helped the country achieve the Millennium Development Goals, they are not demonstrated to be sufficient for preventing mother-to-child transmission of syphilis. The committees’ systematic and bureaucratic agenda has not been efficient in managing avoidable factors for syphilis, nor do they operate in the scope of the integration of surveillance and care actions, as recommended by the health policy.

**Conclusion:**

The committees’ model needs to be reviewed in the context of Brazil's National Health System. The research process should be rescaled in order to remain a cornerstone for the induction of health policy that integrates surveillance and healthcare across Brazilian Federative Units. The advancement toward an automated case management model becomes relevant for the country to meet global commitments to eliminate congenital syphilis transmission and achieve the goals outlined in the 2030 Agenda.

## Highlights

• Prevention of mother-to-child transmission of syphilis.
• Strengthen syphilis control by automating case management with a control strategy.
• Rethink the policy of investigation committees for mother-to-child transmission of syphilis.

## Introduction

Syphilis is a sexually transmitted infection (STI) that represents a global challenge to health systems [1] and society as a whole, as its elimination is a goal yet to be achieved [2,3]. Despite its invisibility, syphilis is one of the leading causes of infant mortality worldwide [4]. Against this background, international entities have launched strategies to control mother-to-child transmission (MTCT) of the disease [5]. The reduction of MTCT cases figures in the list of public strategies for reducing STI-related maternal and infant mortality, especially congenital syphilis and syphilis in pregnancy [1].

The Pan American Health Organization (PAHO) and the World Health Organization (WHO) have been promoting strategies to strengthen public policies in this area. In this context, Brazil and other Latin American and Caribbean countries had agreed to eliminate congenital syphilis by the year 2000 [6], which failed to occur.

In 2006, PAHO, WHO, and the United Nations Children's Fund (UNICEF) launched initiatives to strengthen the prevention of MTCT, HIV and syphilis. During that period, Brazil launched the interfederative policy *Pact for Health*, which reiterated the need to reduce the MTCT of syphilis and HIV [7].

Despite the investment, reducing congenital syphilis is a challenge yet to be addressed. In 2017, PAHO/WHO introduced a proposal to eliminate MTCT of syphilis in the Americas region, with the priority target of reducing the incidence rate of congenital syphilis to ≤0.5 cases per 1000 live births [8].

In Brazil, the “Applied Research for Intelligent Integration Aimed at Strengthening Healthcare Networks for Rapid Response to Syphilis”, also known as the "Syphilis No!" Project was implemented in 2018 with the goal of creating national strategies to eliminate MTCT of syphilis [9], fully aligned with the Sustainable Development Goals (SDGs) of the United Nations (UN) 2030 Agenda [3,10].

As for specific actions to prevent MTCT, Brazil's Ministry of Health (MoH) has induced the policy through a guideline of implementing Committees for Investigation of MTCT (CIMTCT) of syphilis, HIV, and viral hepatitis since 2014. As of 2023, the MoH has continued that induction as a compulsory aspect for municipalities or Federative Units to be certified for the elimination of MTCT (EMTCT) [11,12].

However, the impact of the committee's policy on the epidemiological bulletins of Brazil's National Health System (SUS) cannot be determined at this point. This raises questions on the effectiveness of committees in reducing the incidence of congenital syphilis in Brazil and on the need for such a strategy to achieve the goals set by PAHO/WHO [1].

Meanwhile, although Brazil's MoH established the investigation committees as a key strategy in the policy for prevention of MTCT almost 10 years ago, there is no empirical research examining this tool and its efficiency for policies of syphilis control [13,14].

National and international literature emphasizes the value of identifying gaps in the healthcare network and proposing interventions to reduce syphilis. Hence, this study analyzes the implementation of the committees for investigation of syphilis in Brazilian Federal Units.

### The policy of implementation of committees for investigation of mother-to-child transmission of syphilis in Brazil

In Brazil, the guidelines of investigation committees were framed in the context of implementation of policies to reduce maternal mortality in the 1980s [15]. In that period, committees for investigation of maternal mortality were implemented in the federative units with the MoH financial investment, however, these had autonomy in evaluating their indicators and decision-making for local interventions [16]. These were all fundamental for lowering rates of maternal and infant mortality in Brazil [17].

The success achieved in reducing maternal and infant mortality indicators in Brazil under the Millennium Development Goals led the MoH to propose the very strategy to achieve the SDGs in the UN's 2030 Agenda targeting the reduction of MTCT of STIs [10,18]. However, even though the investigation committees were implicit in the maternal mortality committees' policy, it was only by 2014 that the MTCT investigation protocol was launched as a national SUS guideline. This protocol laid out the recommendations for both investigative processes *per se* and for establishing the investigation committees nationwide [16].

The protocol's release was an important milestone and a core element of the Ministry's political agenda for reducing MTCT of HIV, viral hepatitis B and C, and syphilis in SUS [16]. Through this protocol, the MoH established a stream of support to federative units and municipalities, integrating epidemiological analysis and reflections on the local situation [15].

Nonetheless, in 2016, after an extensive audit, federal control bodies demanded that the Ministry enact more effective measures to address the epidemiological scenario of syphilis in Brazil. This culminated in the 2016 ruling of the Federal Court of Accounts (TCU), which included the reduction of congenital syphilis rates, among other requirements [9].

In the same period, the National Congress approved a parliamentary amendment of approximately 57 million euros. Part of it was decentralized by Brazil's MoH to the Federal University of Rio Grande do Norte (UFRN) in order to support the development of a rapid response project to syphilis in Brazil. As of 2018, that project, known as the “Syphilis No!” Project was incorporated into the Strategic Agenda for Syphilis Reduction, a policy guideline implemented by the Ministry in 2017 [9,19].

In this scope, the “Syphilis No!” Project developed the Healthcare and Surveillance System (Salus) [20], a technological tool integrating surveillance and healthcare actions to fill gaps in the health network through a case management system. Salus works toward achieving the goals laid out to combat syphilis in Brazil, improving the quality of case surveillance, management, and health care. In sum, it is a system that contributes to implementing the Protocol for Investigation of MTCT [16] and advances toward the development of epidemiological investigation actions.

## Methods

The Brazilian territory is divided into five major regions, as follows: **North**—with seven States, i.e., Acre (AC), Amapá (AP), Amazonas (AM), Pará (PA), Rondônia (RO), Roraima (RR), and Tocantins (TO)—, **Northeast**—with nine, i.e., Alagoas (AL), Ceará (CE), Maranhão (MA), Paraíba (PB), Pernambuco (PE), Piauí (PI), Rio Grande do Norte (RN), Sergipe (SE), and Bahia (BA)—, **Central-West**—with the Federal District (DF) and the States of Goiás (GO), Mato Grosso (MT), and Mato Grosso do Sul (MS)—, **Southeast**—with four States, i.e., Espírito Santo (ES), Minas Gerais (MG), Rio de Janeiro (RJ), and São Paulo (SP)—, and **South**—with three States, i.e., Paraná (PR), Rio Grande do Sul (RS), and Santa Catarina (SC). In sum, Brazil encompasses 26 States and the Federal District, Brasília, totaling 27 federative units and 5,570 municipalities, with a population estimated at more than 210 million inhabitants [21].

Within the Brazilian health system, the management of health actions and services is shared between the central level—i.e., the MoH—, the federative units, and the municipalities. In regards to MTCT investigation, the federative unit managers are responsible for conducting actions in collaboration with smaller units.

As the investigation committees’ agenda is an innovation by Brazil's MoH for the whole national territory, we decided to delve into data about the investigation committees of the federative units, which fall under the responsibility of the State Health Secretariats (SHS). Only the State of São Paulo, located in the Southeast region, declined the invitation. This fact did not affect the data analysis since, although São Paulo comprises 645 municipalities, our study covered a total of 4925 municipalities through the managers of all 26 units researched. This represents 88% of Brazilian municipalities and 78% (158,579,541 inhabitants) of the country's population.

The interview technique was conducted by using a semi-structured questionnaire (see Supplementary file) about (i) the implementation and sustainability of the investigation committees and (ii) the work process and importance of federative units’ committees for the syphilis response during 2015-2020.

The research instrument was assessed by three experts (pilot test) who have been practicing in the field of MTCT for over 15 years—all holding a master's or doctoral degree—who evaluated the questionnaire items for consistency, relevance, and comprehension. Moreover, data were collected from November 09, 2021, to January 16, 2022.

The questionnaire was applied to 26 federative units, with 34 representatives from the SHS participating. The inclusion criterion was operating in a federative unit or the Federal District Health Secretariat and actively working in the policy of investigation of syphilis MTCT. All respondents have signed an Informed Consent Form (TCLE, its acronym in Portuguese). On average, the interviews and questionnaire application lasted 90 minutes.

For data analysis, we opted for applying Bardin's content analysis technique [22], a benchmark for qualitative studies on public health policy. Data were analyzed in three phases. Stage (i) involved pre-analysis. Then, stage (ii) comprised exploration of material and results processing. Lastly, stage (iii) was data interpretation, with a detailed analysis of the category's frequency.

This study was approved by three Research Ethics Committees (REC). Therefore, the REC of the Onofre Lopes University Hospital, at the Federal University of Rio Grande do Norte (REC/HUOL/UFRN), issued the Certificate of Presentation for Ethical Consideration (CAAE) No. 50254021.0.0000.5292.

## Results

### Characterization of the investigation site of mother-to-child transmission by federative unit

Although the investigation committees represent a national policy guideline, not every Brazilian Federative Unit uses this term to characterize the investigative work developed in its own institutional space. Despite most SHS (n = 15; 58%) adopting the terminology CIMTCT of syphilis, in n = 11 federative units, the syphilis investigation work has not been characterized as that of a committee. It can be from the other institutional spaces such as STI Coordination, the Committee Maternal Mortality, the Surveillance Coordination, from Syphilis Working Groups, and the Infant Death Committee. It very often can be located in Primary Health Care (PHC) and health surveillance—notably, the Alagoas Federative Unit.

**Figure 1:**
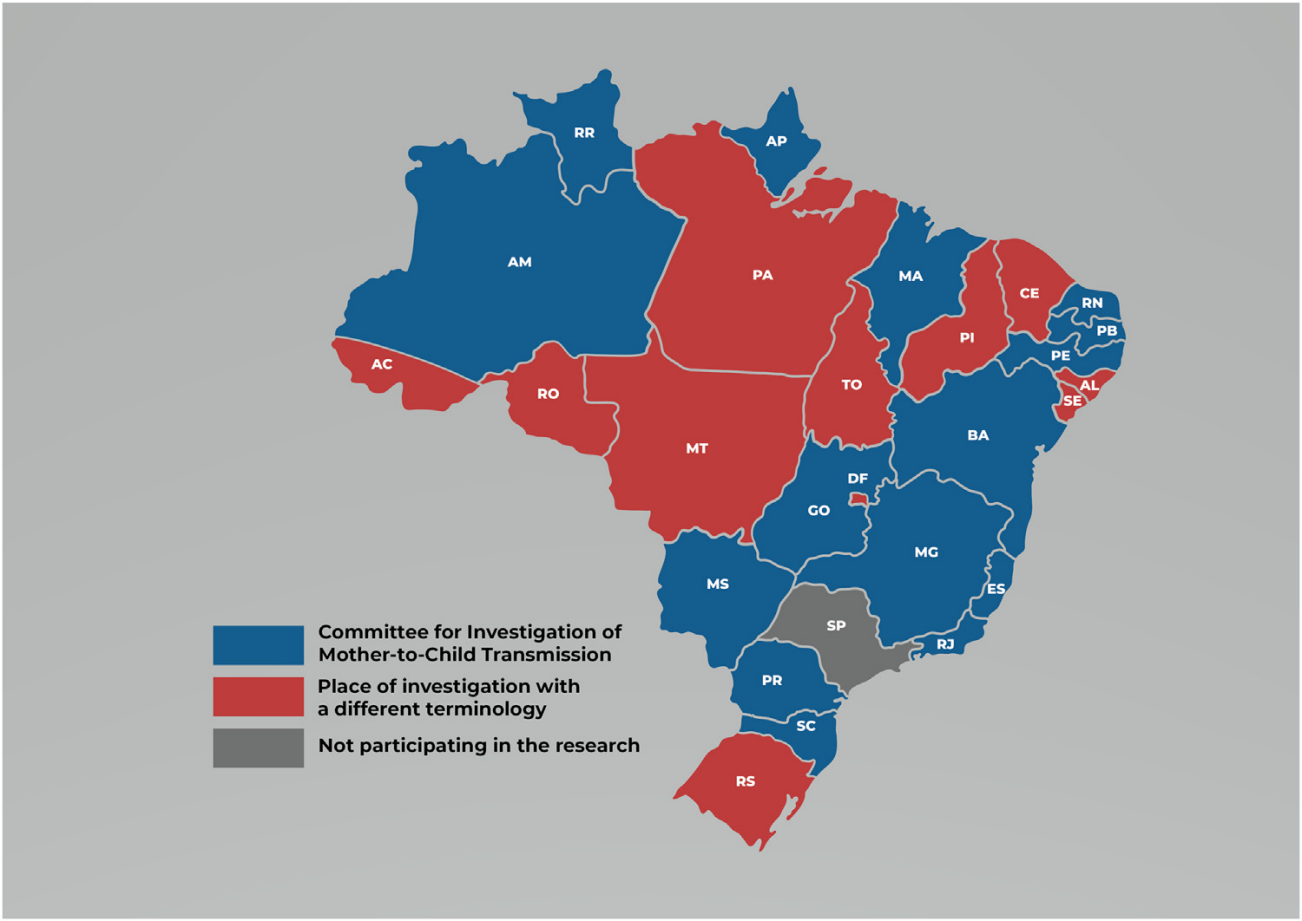
Existence of a committee or other institutional space for the investigation of diseases transmitted from mother to child, Brazil, 2022. Source: own authorship, 2022.

Figure 1 breaks down the distribution of such terminology per federative unit. It is worth noting the implementation of investigation committees of nine federative units—AM, BA, MA, MG, PR, PA, PE, RN, and RO—initiated in 2018. Meanwhile, in the federative units of AP, ES, GO, MS, RJ, and SC, the investigation committees were implemented between 2015–2017. Nevertheless, not all of them addressed the issue of syphilis in their agenda, which is the case of AP.

### Inducing the implementation of committees for investigation of mother-to-child transmission through the “Syphilis No!” project

As of 2018, there was a significant rise in the implementation of investigation committees, which was the case for n = 15 (57.6%) federative units, especially in the Northeast and northern regions of Brazil. This includes the creation of specific committees for syphilis and the inclusion of the syphilis agenda in other places that had already been conducting investigations for notifiable diseases. That is, as of 2018, the actions of the “Syphilis No!” project had a significant impact on the investigation agenda concerning syphilis MTCT in n = 21 (80.7%) federative units, especially for the inclusion of the topic in places that had already been investigating other notifiable diseases.

Furthermore, the project has contributed to supporting actions for preventing syphilis nationwide, promoting continuing education, undertaking information and communication actions, and strengthening the integration between primary care and health surveillance in all Brazilian regions.

### Implementation of investigation of mother-to-child transmission of syphilis in investigation committees of federative units

In this study, we deemed as “investigation of MTCT of syphilis implemented in the investigation committees of the federative units” the cases when a respondent confirmed that syphilis was on the committees’ programmatic agenda, that was because, in some situations, the committees were defined as a policy of MTCT prevention, and while some progress was made regarding the configuration of a general committee, syphilis was not included in their investigation agendas.

**Figure 2:**
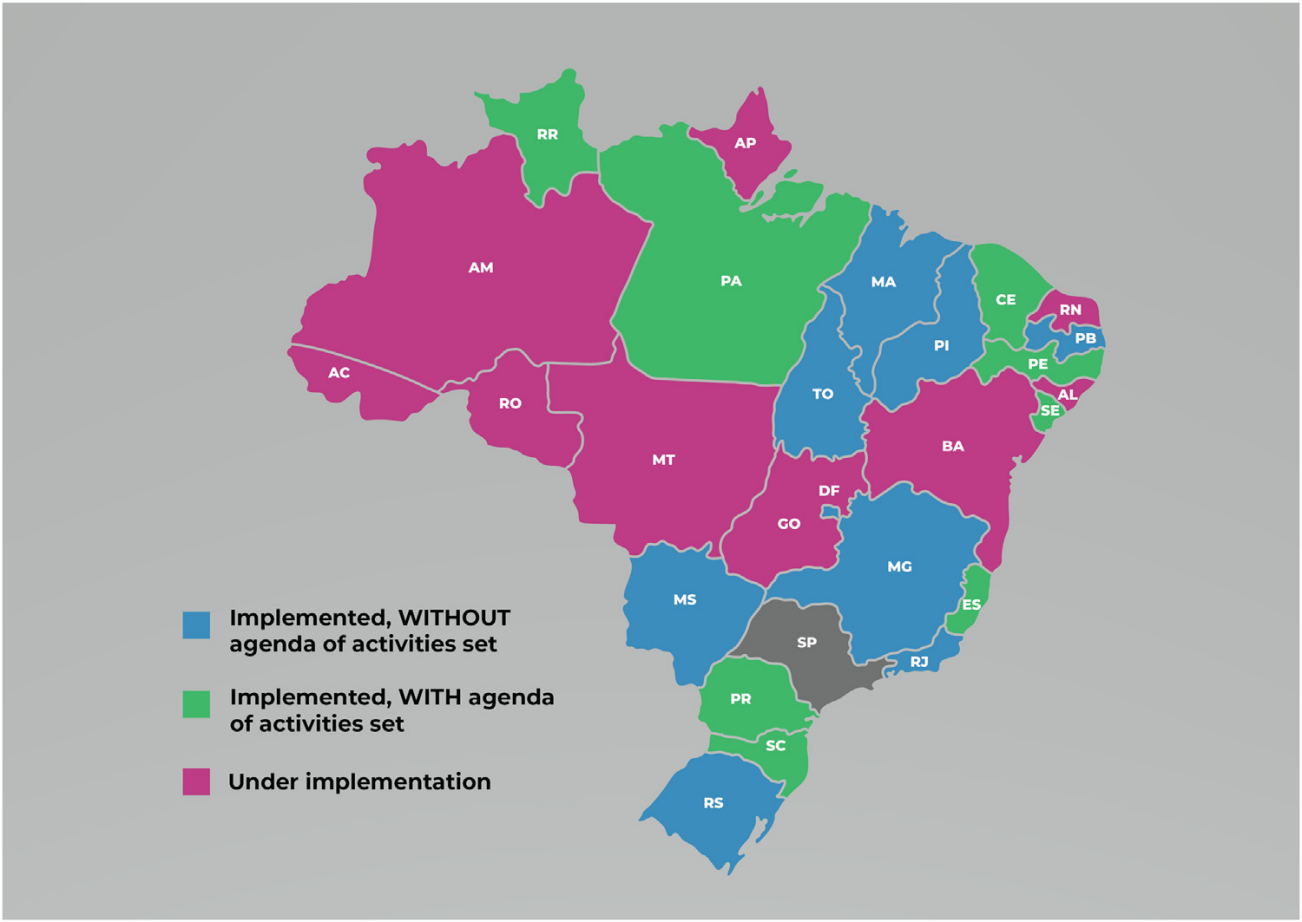
Status of the institutional spaces responsible for performing investigations of mother-to-child transmission of syphilis, n = 26.

In light of this, considering the functioning of the committee's agenda for syphilis in federative units that have established the committees, we observed that n = 9 (35%) had not included syphilis in their agendas, and n = 8 (30%) have fully implemented syphilis investigation, with a set agenda of activities (see Figure 2). Moreover, it was found that the Central-West, Northeast, and North regions have a kind of institutional space for investigating MTCT of syphilis under implementation, as they have not yet formed committees entirely in line with the MoH guidelines. On the other hand, Brazil's Southeast and South regions have committees compliant with the policy guideline, while not every federative unit has syphilis included in their agendas.

Data also reveal that the establishment of the investigation committees in line with the Ministry guidelines, as well as the inclusion of syphilis in the investigation agenda, is not the main factor determining whether investigations are carried out. In effect, interviewees reported that some places had been formally implemented without an agenda of activities to be followed and, even so, investigating cases independently of the institutional agenda—e.g., RS, MA, TO, DF, PI, RJ, MG, and PB. In contrast, other places have signaled they have defined an agenda of activities but have not been holding meetings to investigate cases, such as the federative units of PA and SC.

### The role of investigation committees in investigations of mother-to-child transmission of syphilis

The interviewees emphasized that the improvement of the healthcare network, the investigation of syphilis MTCT cases, the integration between assistance and surveillance, and the standardization and regulation of healthcare network flows fall under the responsibilities of the committees in operation. Regarding the data on committees that do not fulfill their role, the interviewees reported that one of the reasons is the shortage of local resources, particularly personnel, as well as the lack of implementation of systematic actions or the need to formalize technical cooperation with municipalities. These facts hinder the agenda for establishing and strengthening the investigation committees.

### Conformation of committees and responsibilities for investigation of mother-to-child transmission of syphilis

**Figure 3:**
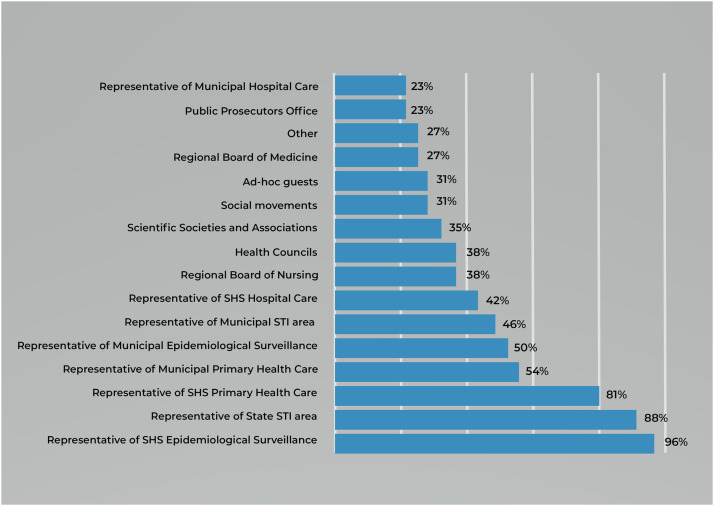
Percentage of composition of members that are part of the place for investigation of syphilis mother-to-child transmission. n = 26. SHS, State Health Secretariats; STI, sexually transmitted infection. Source: own authorship, 2023.

Figure 3 gives the distribution of professionals responsible for MTCT investigation of syphilis in committees. In n = 25 (96%) federative units, except for TO, there is representation of surveillance professionals from the State Health Secretariat. In n = 23 (88%), there are professionals in the field of STI infections from the State Health Secretariat, except for SE, RJ, and RO. Lastly, in n = 21 (81%), there is representation of the PHC coordination area in the State Health Secretariat, except for AM, BA, MT, RS, and TO.

**Figure 4:**
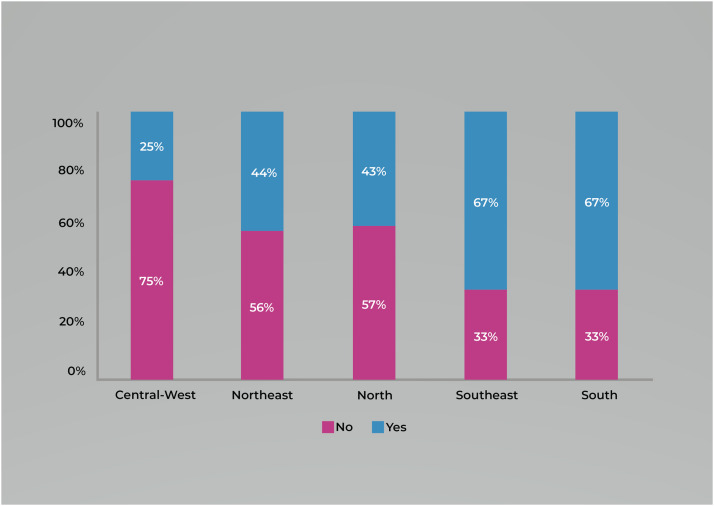
Percentage of participation of the project supporter as a member of a place responsible for the investigation of mother-to-child transmission along the project's territorial support per region. n = 26. Source: own authorship, 2023.

Throughout the project's execution period, a team of professionals assigned as Research and Intervention Supporters worked at the local level to meet the goals to prevent MTCT of syphilis. In some federative units, these professionals have served as short-term members of the investigation committees. Figure 4 depicts the percentage of participation of such professionals in the committees. In the South and Southeast regions, n = 2 (67%) federative units were assisted by the supporters. This was followed by the Northeast, North, and Central-West regions, with n = 4 (44%), n = 3 (43%), and n = 1 (25%), respectively.

It should be noted that although there are representations of several professionals in the committees investigating syphilis MTCT, n = 14 (54%) of interviewees reported there was no flow defined for members to join or leave.

### Agenda and work process of committees in the investigation of syphilis mother-to-child transmission in the federative units

The results show that n = 16 (62%) federative units conduct periodic meetings to discuss the investigation of syphilis MTCT, either once a month (n = 9, 35%); once a bimester (n = 5, 19%); twice a month (n = 1; 4%); or once a week (n = 1; 4%). Meanwhile, n = 10 (38%) respondents stated that no meetings were occurring. The main reasons reported for not holding meetings were disorganization, non-composition of group members as the MoH recommends, unavailability in members' schedules, or other competing priorities, such as the recent COVID-19 pandemic.

**Figure 5:**
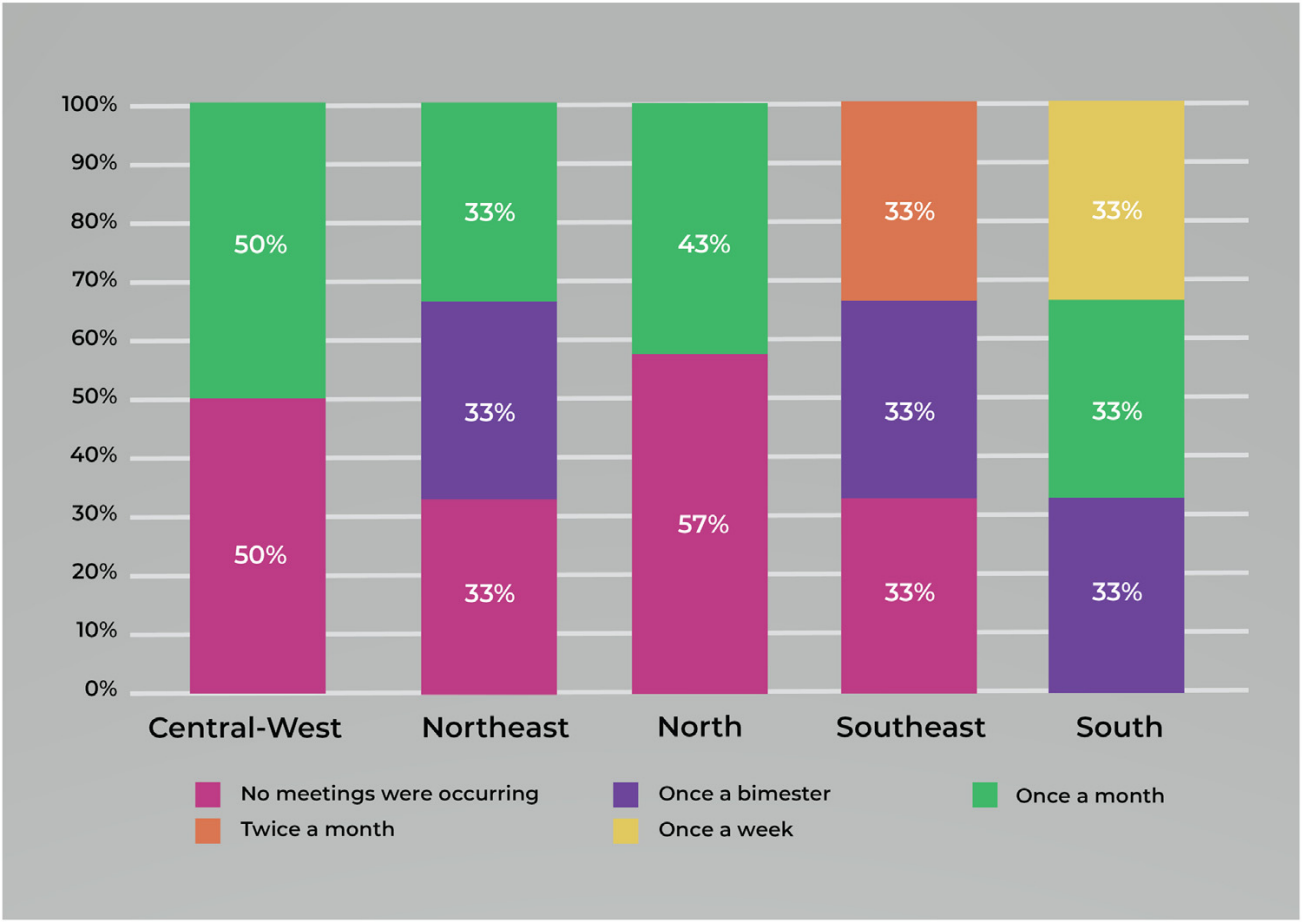
Percentage of meeting periodicity coordinated by the institutional spaces for mother-to-child transmission of syphilis per region. n = 26. Source: own authorship, 2023.

The South region is the only one in the country where all the federative units’ spaces destined to investigate the cases have been holding meetings periodically. Conversely, the Central-West and North regions have, respectively, n = 2 (50%) and n = 4 (57%) federative units in the opposite situation, i.e., no meetings are occurring. The same goes for the Northeast and Southeast regions, where n = 3 (33%) and n = 1 (33%) federative units from both have not been hosting meetings (Figure 5).

Only a few cases of syphilis were investigated, and when they were, there was little or no feedback to the healthcare network, where one can note the lacking or poor monitoring of syphilis cases, especially in children with congenital syphilis.

### Integration between primary care and health surveillance areas of Brazil's National Health System

All the federative units signaled that integration with the healthcare network, especially between primary care and health surveillance services, is fundamental in enhancing local interventions performed by the committees for investigation of syphilis. However, it was observed that the committees— which should investigate the MTCT of syphilis—have not effectively performed this integration, particularly because they do not promote articulation between assistance and surveillance in Brazil. In sum, we identified fragilities in the committees’ role of strengthening case management in the healthcare network.

Data demonstrated such poor integration as a national challenge for MTCT case management due to a lack of articulation and systematization of the work process among Brazilian regions. On top of that, the shortage of human resources, the competing priorities in the federative units’ agenda, and the lack of infrastructure and care sharing system are some of the reasons behind this challenge.

## Discussion

The MTCT investigation protocol by Brazil's MoH presents the committees for investigation of syphilis as institutional spaces responsible for investigating the disease outcomes in Brazil [16]. However, it became evident that the practice of investigating syphilis MTCT has not necessarily been in line with the Ministry's norms and regulations. Within the health management of federative units, that practice occurs in various institutional spaces, demonstrating the decentralization of investigations and adaptation of such spaces to the realities of federative units regardless of the Ministry's protocol.

As several studies have shown [13,23], organization and work processes are multiple and specific to each federative unit in the five regions of Brazil. In addition, evidence was found that the dynamics and criteria used to investigate and manage syphilis cases in the different federative units also vary. One hypothesis that could explain such a finding is that the institutional protocol for implementing committees in a systematic and bureaucratic meeting format may hinder research, investigative agendas, and actions—since the federative units’ idiosyncrasies comply with other work dynamics, which in most cases do not bear the joint analysis and forwarding of hundreds of notification forms. Such a practice should require automation.

The reasons why the federative units adopt different types of committees, organizations, and work processes could be linked to their particular histories of implementing policies in SUS. Varied social and cultural contexts and diversities could also explain these differences [24].

Moreover, all interviewees underscored the importance of investigation spaces in the analysis of preventable events and diseases, the identification of intervention measures, and the enhancement of the healthcare network. However, investigation committees designated by federative units for investigating syphilis cases have not fulfilled their primary role of investigating cases. In those cases where the practice has been performed, little or no feedback was offered to the healthcare network.

There are low follow-up of syphilis cases, especially among exposed children or those with congenital syphilis. Brazil's MoH Ordinance No. 3276 determines that financial resources can only be transferred to a federative unit through systematic and regular follow-up of surveillance actions [19,25]. MoH funding policy may be transferring resources to federative units that report cases of congenital syphilis without due outcome and consequent notification. Moreover, the establishment of an investigation committee, *per se*, does not ensure that investigation of cases is a topic in the case investigation agenda.

According to Borges [15], an adequate flow of incoming and outgoing members in investigation committees is essential for ensuring their long-term sustainability. However, this flow has been virtually nonexistent in the committees, which may render it even more challenging for the continuity and sustainability of such institutional spaces. Besides, there is a risk of the committee becoming deserted and, consequently, unsustainable.

The integration between health and surveillance fields is widely discussed in national and international literature as fundamental to advancing health agendas. In Brazil, it has been proposed that, in a joint and coordinated manner, the two fields work to identify causes, outcomes, and more effective decision-making, especially in the investigative process of syphilis MTCT in federative units [26,27].

However, data relative to investigation committees revealed that integration between areas has been challenging for federative units due to a shortage of human resources, a lack of prioritization of the issue by the federative units, and a scarcity of automated systems to manage cases of syphilis.

The national and international body of literature, including technical documents from Brazil's MoH, stress that public health policies are a shared responsibility in the different dimensions of health systems management and must be fostered and expanded within strategic agendas. In syphilis response, researchers demonstrated that in Brazil the “Syphilis No!” Project has been a relevant tool for strengthening it since 2018 [9,^27^,28].

Brazil's MoH does not currently disclose in its bulletins the number of investigated, discarded, and confirmed cases, especially concerning congenital syphilis notifications. This lack of information hampers the analysis of the reduction of syphilis MTCT and the effectiveness of health policies. Almeida *et al.* [3] emphasized that the absence of data for monitoring and surveillance is a significant gap hindering the evaluation of actions and strategies developed in the healthcare network. It is urgently necessary to gather data for more assertive decision-making. Indeed, for assertive reporting of congenital syphilis cases [19].

The “Syphilis No!” created the Healthcare and Surveillance System, namely Salus, a platform for the automated management of syphilis cases and for intelligent monitoring of diseases in healthcare and surveillance [29]. Some municipalities are currently implementing this system, as Salus has already been pointed out as an important tool for analyzing avoidable factors in syphilis control through case management. That is possible because Salus combines surveillance and healthcare data that can be shared in real time across the various services and professionals along the healthcare network. Such a strategy can strongly contribute to reducing the fragility of Brazil's surveillance systems [20].

Health policies decentralization and their capillarization to SUS services is part of the interfederative management configuration in Brazil. Moreover, such decentralization underscores the role of federative units' administration in developing surveillance and comprehensive healthcare actions [15]. Our study points to the need to reassess the practices for syphilis cases investigation, especially at a federative unit level, and reiterates the demand for integration between healthcare and health surveillance services. Under the current conformation induced by Brazil's MoH, the investigation committees do not fulfill the demands of integrated practice.

In this vein, the Ministry must evaluate the cost-effectiveness of continuing the committees as a SUS policy, as it requires a major investment in the improvement of work processes, the retention of professionals in the committees, and sustainability of investigation. The adequate monitoring of syphilis cases is primordial to achieving the prevention of unfavorable outcomes [30].

## Conclusion

Our results revealed that even though the syphilis investigation committee is a policy defined as a priority in SUS, there was no national consolidation according to its proposal. This is because the model designed for committees has proven to be unviable.

In this context, the research states the importance of the “Syphilis No!” Project in shaping the Ministry policy on syphilis, as well as in promoting and improving the healthcare network through the epidemiological investigation of cases across Brazil. The project endeavored to include syphilis in the investigation committees’ agendas and to foster preventive actions, integration between assistance and surveillance, continuing health education, and information dissemination and communication, relying on institutional support.

Despite syphilis being prioritized in the management agendas of Brazil's health system, more effective and pragmatic actions are still needed, focusing on integration between surveillance and health care. It is, therefore, essential to model the actions of the committees investigating MTCT of syphilis in the context of digital health, such as by adhering to the Salus platform. Our findings indicate that one plausible reason for the unfeasibility of the MTCT investigation committees is their cost-effectiveness and overall impact on the policy. This could be attributed to the fact that digital health solutions have not been incorporated.

This study found a lack of case monitoring of children exposed to syphilis. Each federative unit has multiple and specific work processes, dynamics, and criteria for investigating and managing syphilis cases. The differences range from the implementation of services to the organization of agendas and work processes, including the focus placed on the role of these institutions and the flows of mobility of members of spaces for investigation.

Our research on the investigation committees presents a critical reflection on the investigation of cases as an integrating component of surveillance and healthcare actions. Moreover, it reaffirms the significance of shared management among the federative units for health policy implementation.

WHO highlights that one of the criteria for certifying the certified for elimination of MTCT of syphilis is for the country to have effective case management systems for infectious diseases. Meanwhile, Brazil's MoH highlights the importance of the systematic and regular monitoring of cases in order to transfer funding to the federative units. However, regarding the integration between surveillance and healthcare, the investigation of syphilis cases in Brazil could be more effective and efficient if the Ministry actually implemented health information systems, such as Salus, to improve prenatal and postnatal case management. Currently, this fact does not occur in Brazil, as the health information systems of the Brazilian MoH are fragmented and obsolete.

Nevertheless, only the most difficult cases should be pointed out for assessment and opinion by the investigation committees in order to increase efficiency in the use of this highly specialized analysis. The CIMTCT could assume the role of reviewing the Clinical Protocol and Therapeutic Guidelines (CPTG) for comprehensive care for people with STIs, particularly in relation to congenital syphilis and syphilis in pregnancy, as the protocol requires regular adjustments. For instance, CPTG's latest update recommends notifying a suspected case of congenital syphilis even in case of doubt, whereas this should only occur after investigation—such inaccuracy could contribute to an unrealistic scenario of the syphilis epidemic in the country.

We encourage further studies on the subject to delve deeper into the organization and work processes of investigation spaces. In addition, their main challenges and potentials should also be studied, notably for countries that have already eliminated syphilis, such as Cuba.

It should be noted that the ineffectiveness of committees in investigating MTCT hinders the implementation of the national policy for syphilis response, as it undermines epidemiological analysis and timely monitoring of the syphilis epidemic, declared in 2016.

## Declarations of competing interest

The authors have no competing interests to declare.
